# The Effect of Abobotulinum Toxin A on the Symptoms of Raynaud’s Phenomenon: A Case Series

**DOI:** 10.7759/cureus.8235

**Published:** 2020-05-22

**Authors:** Amelia R Winter, Kathlyn Camargo Macias, Sun Kim, Naveed Sami, David Weinstein

**Affiliations:** 1 Internal Medicine, University of Central Florida College of Medicine, Orlando, USA; 2 Dermatology, University of Central Florida College of Medicine, Orlando, USA

**Keywords:** abobotulinum toxin a, botulinum toxin, raynaud disease, case series

## Abstract

Raynaud’s phenomenon (RP) is a relatively common syndrome occurring alone or in combination with autoimmune and inflammatory diseases. It is characterized by pain and ulceration due to vasospasm in response to cold and stress, most often affecting the digits. Although pharmacologic treatment for this condition exists, it is not always efficacious. Our case series demonstrates the use of abobotulinum toxin A in the treatment of RP. We report the cases of four patients who received injections of abobotulinum toxin A to treat their mild to severe RP symptoms. They experienced clinical improvement for up to one year after treatment.

## Introduction

Raynaud’s phenomenon (RP) is an exaggerated vascular response triggered by ambient cold and emotional stress. Primary RP (Raynaud’s disease) occurs without comorbidities while secondary RP (Raynaud’s syndrome) is associated with an underlying collagen vascular disorder [1]. RP affects over 90% of patients with scleroderma as well as over 10% of patients with systemic lupus erythematosus, dermatomyositis, rheumatoid arthritis, and primary Sjӧgren’s syndrome [2]. Additionally, secondary RP occurring in immunological and rheumatic diseases can lead to amputation of the affected digits [2]. Clinical manifestations of RP occur as a result of vasospasm of the digital arterial circulation, but can also affect the nose, ears, and nipples. When the vasculature is triggered, a “Raynaud’s attack” occurs, causing the affected skin tissue to first turn white, followed by blue due to a lack of oxygenation, and finally red as circulation is restored. A decrease in arterial circulation leads to ischemia, causing pain and functional limitations as well as ulcers and gangrene [3]. This can prove extremely debilitating to patients and reduces their quality of life. Treatment options include medications, such as calcium channel blockers, phosphodiesterase inhibitors, and topical nitroglycerin, surgery, and amputation [4,5]. The specific use of botulinum toxin (BoNT) for the treatment of RP is not approved by the FDA [6]. However, studies have shown that BoNT reduces symptoms in certain patient populations with RP [1,7-9].

Produced by the Clostridium botulinum bacterium, there are seven subtypes of the botulinum neurotoxin (A, B, C1, D, E, F, and G), with only types A and B used clinically [10]. BoNT works as a vasodilator by blocking the release of the neurotransmitter acetylcholine and thereby preventing muscular contraction [10]. There are several different commercially available versions of type A BoNT, such as onabotulinum toxin A, abobotulinum toxin A, incobotulinum toxin A, and prabotulinum toxin A. Despite showing similar efficacies, studies have shown that one unit of onabotulinum toxin A to three units of abobotulinum toxin A is an appropriate treatment conversion, which must be taken into consideration during preparation [11]. Although approved by the FDA to treat muscle spasms, the specific use of BoNT for the treatment of RP is not recognized [12].

We discuss the case of four patients who were treated with abobotulinum toxin A in various anatomic areas affected by RP; they reported clinical improvements in their pain as per a visual analog scale (VAS) and subjective reporting of other symptoms, such as weakness.

## Case presentation

Methods

**Figure 1:**
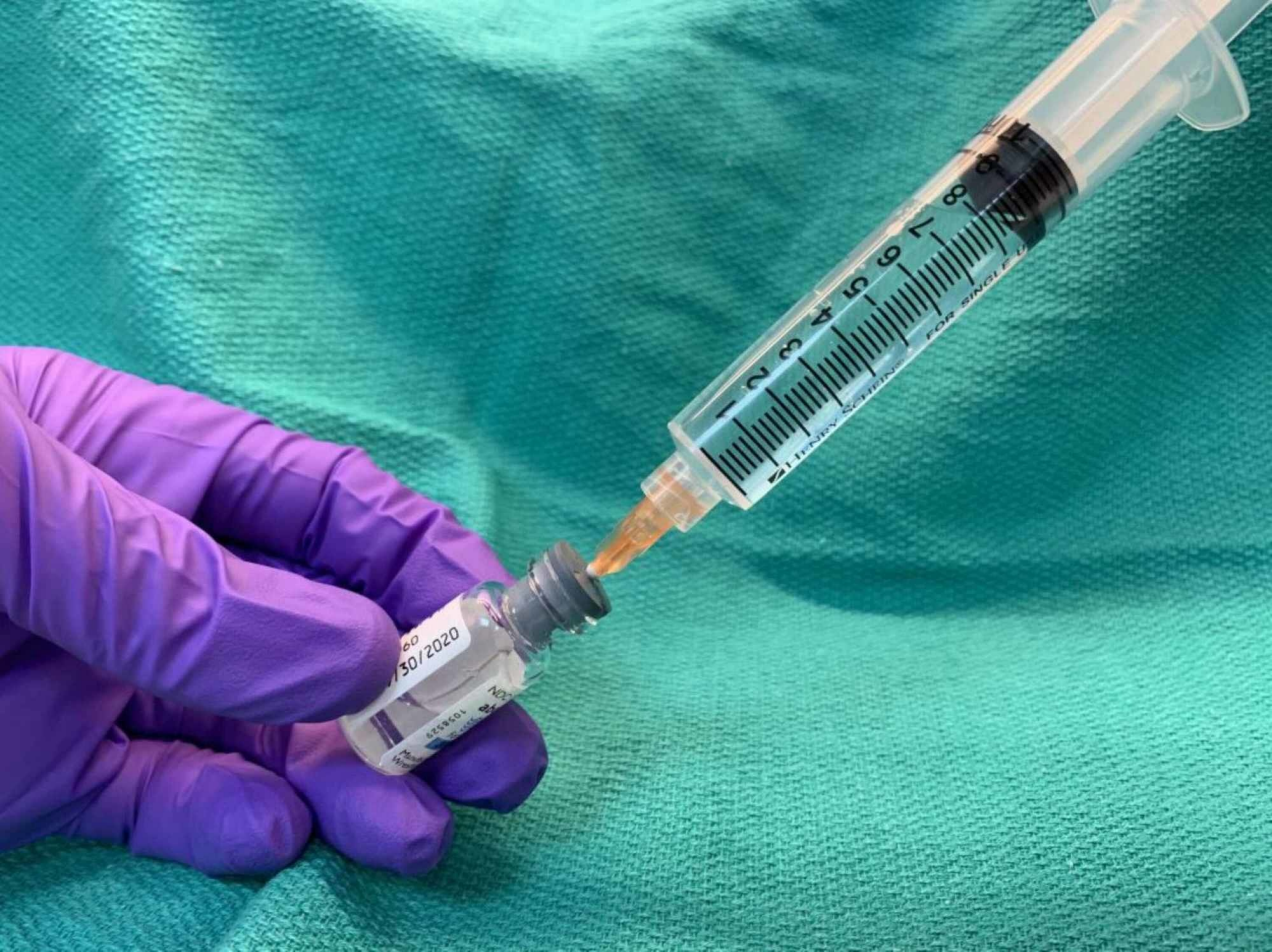
Demonstration of solution preparation

Patients were injected with a prepared solution of onabotulinum toxin A reconstituted with lidocaine or preservative-free normal saline to a concentration ranging from 24 units/1 ml to 150 units/1 ml (Table 1). The volume of diluent needed for injection was drawn up with a 25-g 1-inch needle into either 1-ml, 3-ml, 5-ml, or 10-ml syringe depending upon the desired volume and concentration needed for treatment (Figure 1). Then, approximately 1-2 ml of the diluent was injected into the vial of the abobotulinum toxin without removing the syringe. This was then aspirated back into the syringe. The syringe and needle were then removed from the vial and the 25-g needle was replaced with a 30-g ½-inch needle and gently tilted side to side to evenly mix the solution. Before each injection, ice in an exam glove was placed on the injection site for five seconds to help diminish the pain of injection.

**Table 1:** Summary of treatment and symptom change in patients *As of writing this paper VAS: visual analog scale

Patient	Location	Type of toxin	Number of units injected	Number of points of Injection	Concentration (units/ml)	Diluent	Number of repeat dosages per year	Symptom change	Average duration of improvement
1	Right hand	Abobotulinum toxin A	300	10	30 units/1 ml	Saline	0	Notable weakness within the first 6 weeks of treatment that resolved; since 9 months post-treatment, she has continued improvement of her symptoms (e.g., improved skin quality)	3 months
2	Right hand	Abobotulinum toxin A	300	10	30 units/1 ml	Saline	0	Notable weakness within first 6–8 weeks of treatment that resolved; by 12 months post-treatment, she has continued improvement of her symptoms (e.g., less pain)	3 months
3	Right hand	Abobotulinum toxin A	300	10	30 units/1 ml	Lidocaine	2	Overall hand pain decreased from 8 to 2 out of 10 on VAS for pain, moderate weakness	3.5 months
		Abobotulinum toxin A	270	10	54 units/1 ml	Lidocaine	0	Decreased hand pain, mild weakness	*3 months
		Abobotulinum toxin A	240	10	24 units/1 ml	Lidocaine	0	Decreased hand pain, minimal weakness	10 months
	Left hand	Abobotulinum toxin A	240	10	24 units/1 ml	Saline	0	Decreased hand pain	4 months
		Abobotulinum toxin A	270	10	54 units/1 ml	Lidocaine	0	Decreased hand pain, mild weakness	4 months
4	Left hand	Abobotulinum toxin A	300	10	30 units/1 ml	Lidocaine	0	Decreased pain and skin induration, moderate weakness	13 months
	Left thumb	Abobotulinum toxin A	120	4	60 units/1 ml	Lidocaine	0	Decreased pain	6 months*
	Right hand	Abobotulinum toxin A	300	10	30 units/1 ml	Lidocaine	0	Decreased pain	13 months
	Right index finger	Abobotulinum toxin A	120	4	60 units/1 ml	Lidocaine	0	Decreased pain	6 weeks
	Right index finger	Abobotulinum toxin A	60	4	60 units/1 ml	Lidocaine	0	Decreased pain	5 months*
	Right ring finger	Abobotulinum toxin A	120	4	60 units/1 ml	Lidocaine	0	Decreased pain	11 months*
	Right fifth finger	Abobotulinum toxin A	60	4	60 units/1 ml	Lidocaine	0	Decreased pain	3 months*
	Left foot	Abobotulinum toxin A	210	6	30 units/1 ml	Lidocaine	0	No pain in toes within 1 month of injections	9 months
	Left great toe	Abobotulinum toxin A	45	10	150 units/1 ml	Lidocaine	0	Decreased pain and skin induration	6 months*
	Left fifth toe	Abobotulinum toxin A	60	10	30 units/1 ml	Lidocaine	0	Decreased pain and skin induration	6 months*

**Figure 2:**
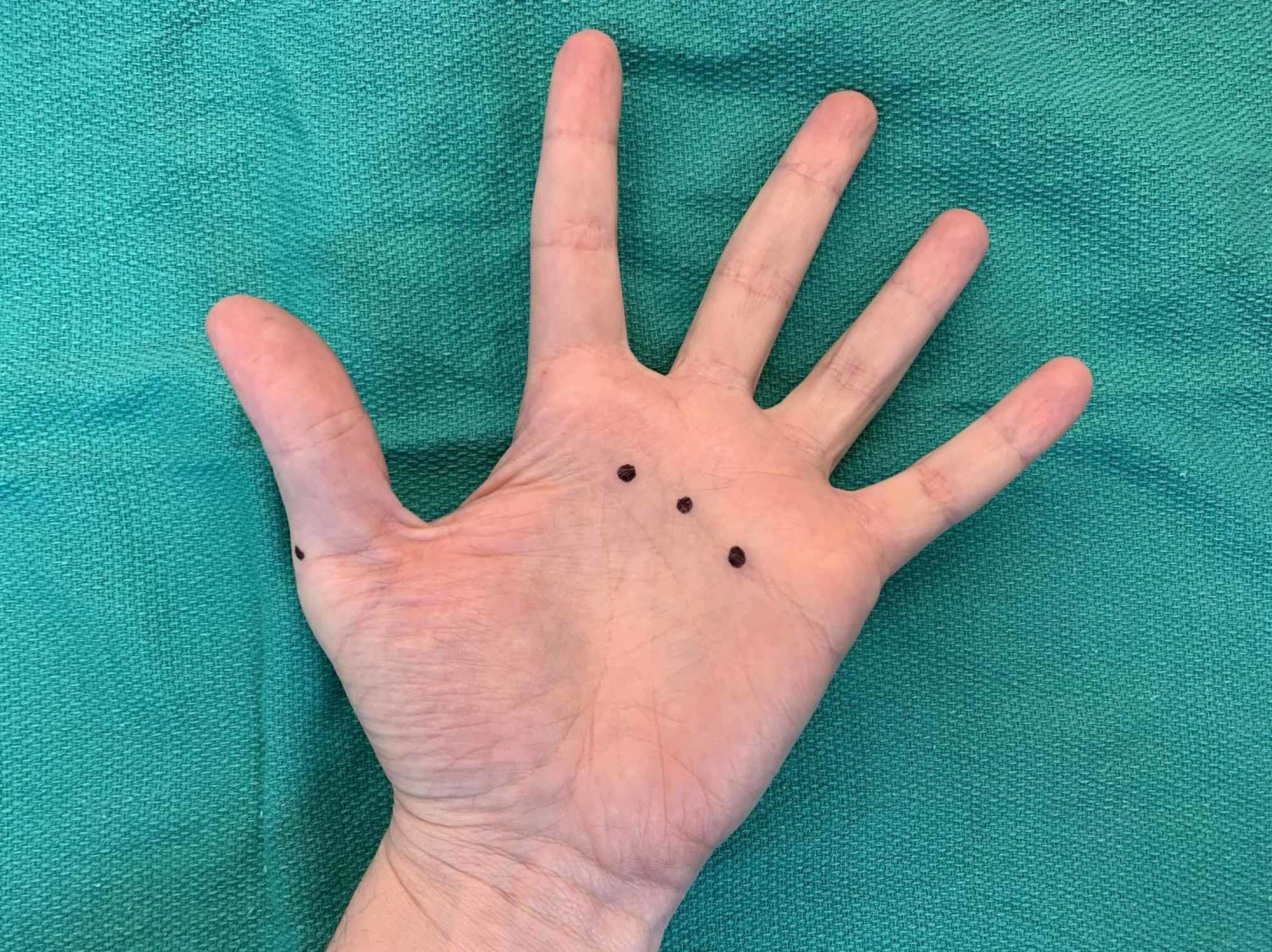
Example of palmar and radial side of first digit injection sites as denoted by black dots (left hand)

**Figure 3:**
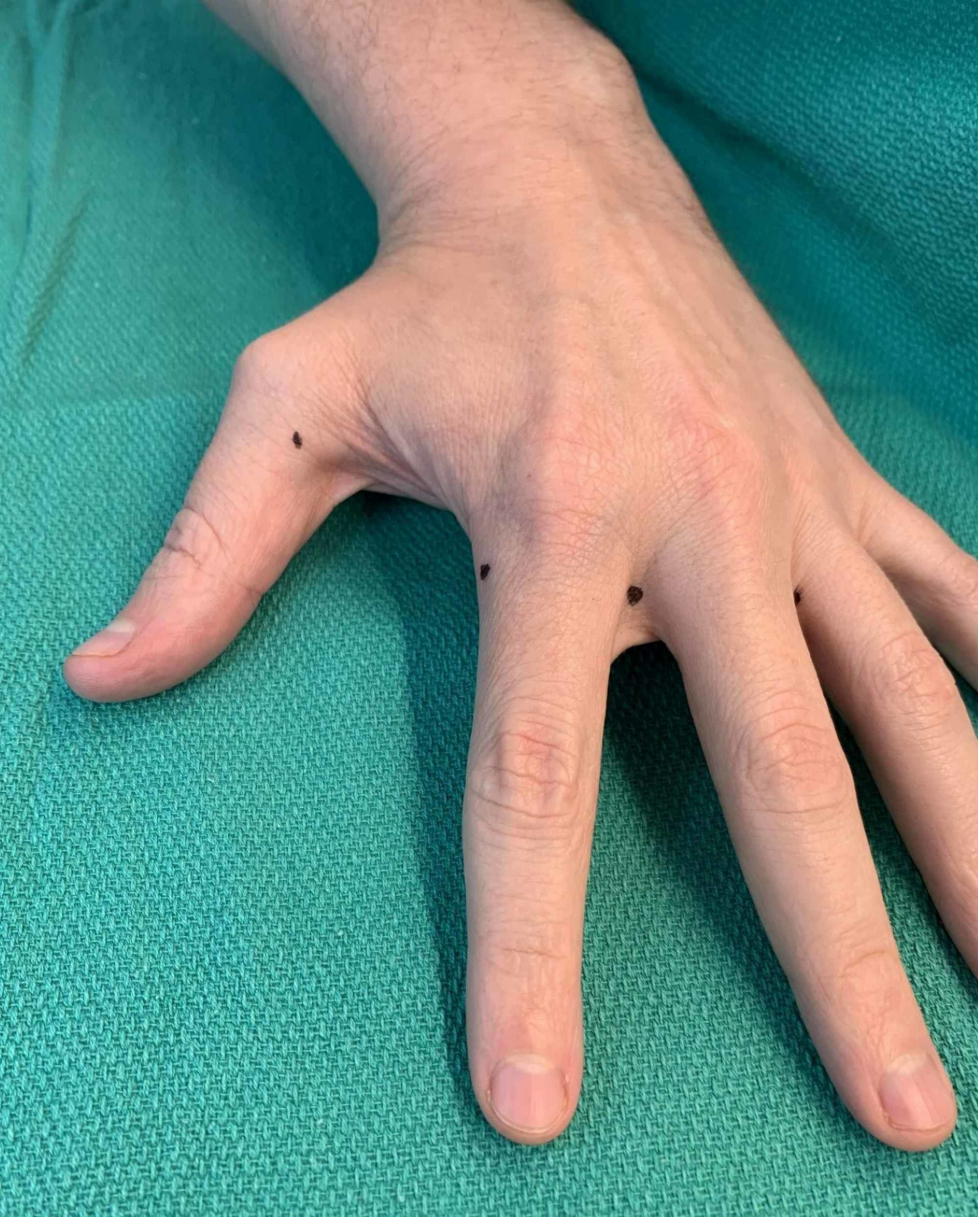
Example of first digit, second digit, and webspace injection sites as denoted by black dots (left hand)

**Figure 4:**
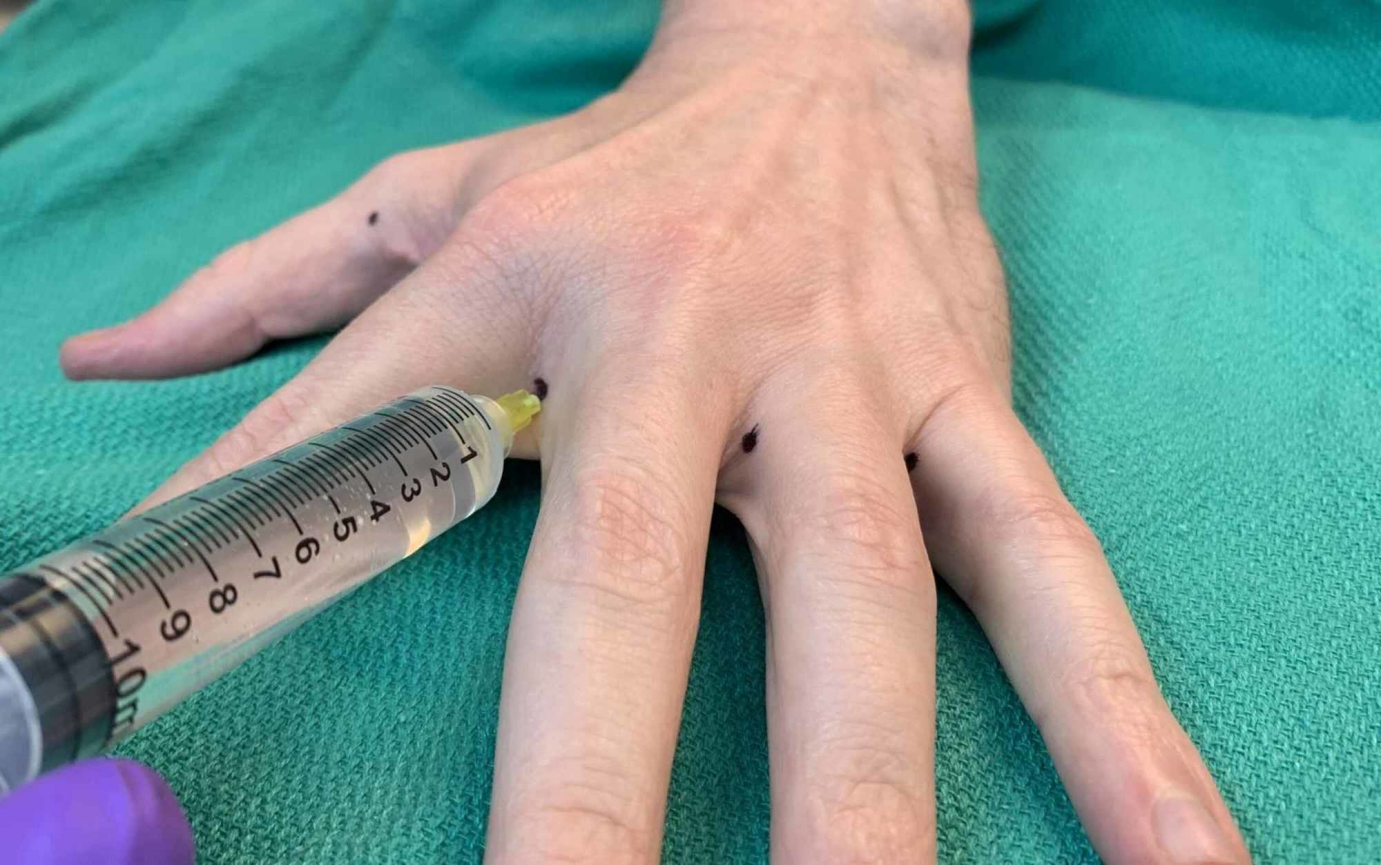
Demonstration of webspace injection technique (left hand)

**Figure 5:**
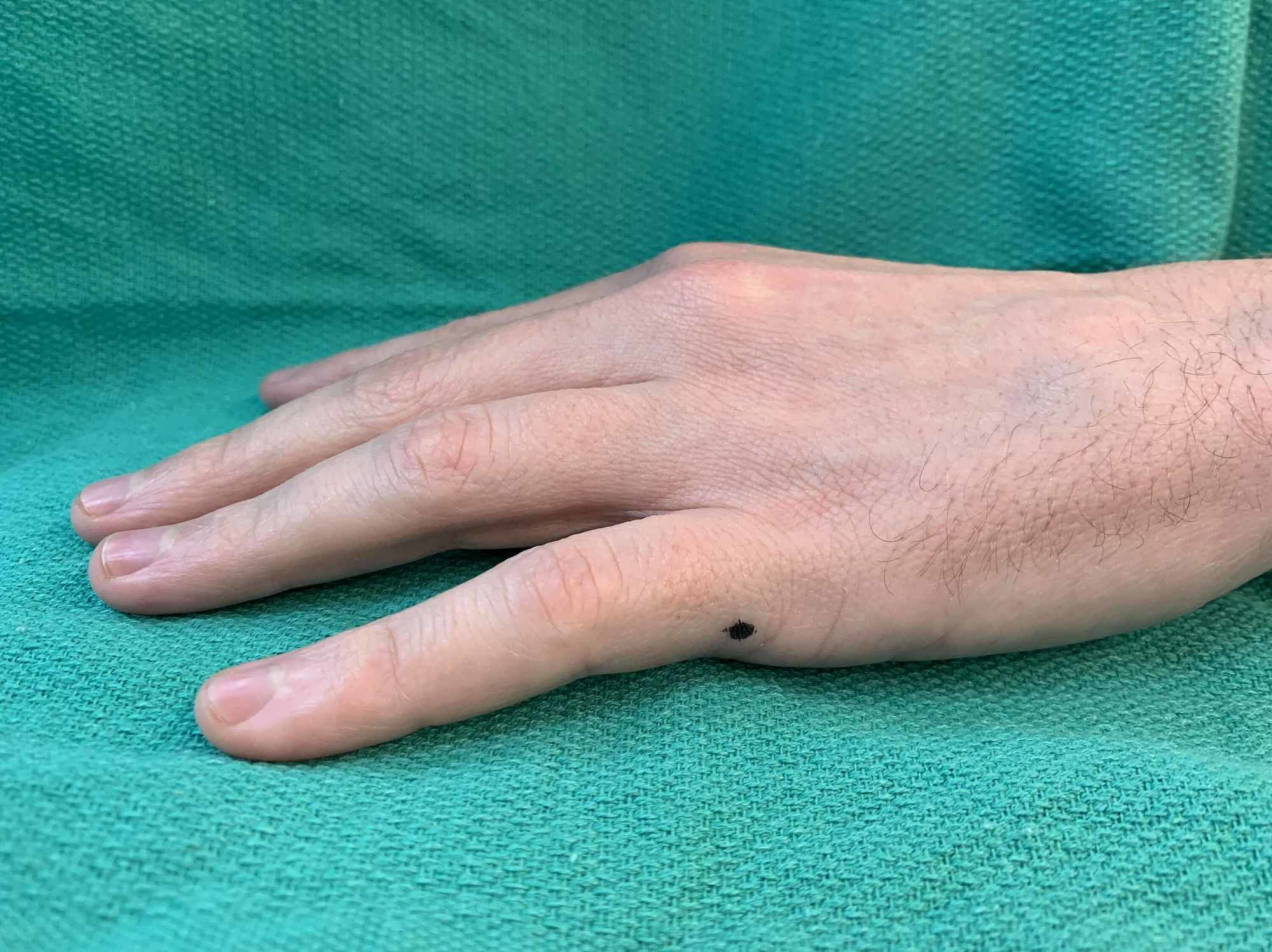
Example of the fifth digit injection site on the ulnar side as denoted by a black dot (left hand)

**Figure 6:**
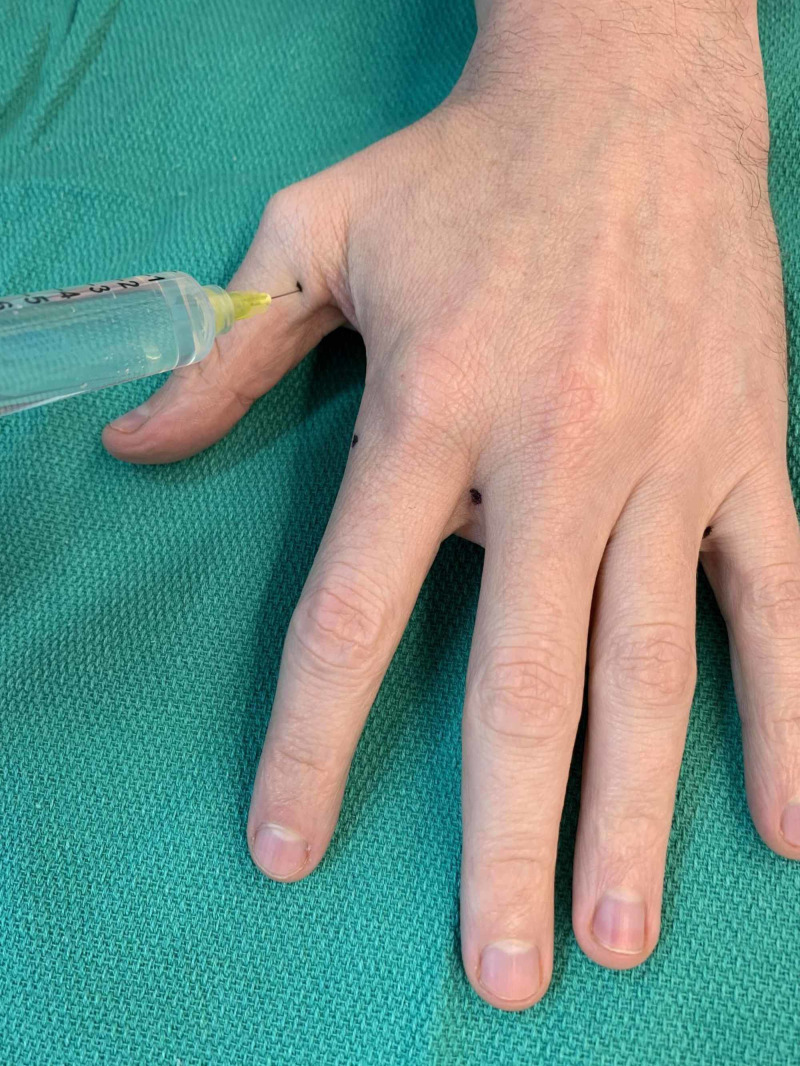
Demonstration of injection technique at the base of the first digit on the ulnar side (left hand)

**Figure 7:**
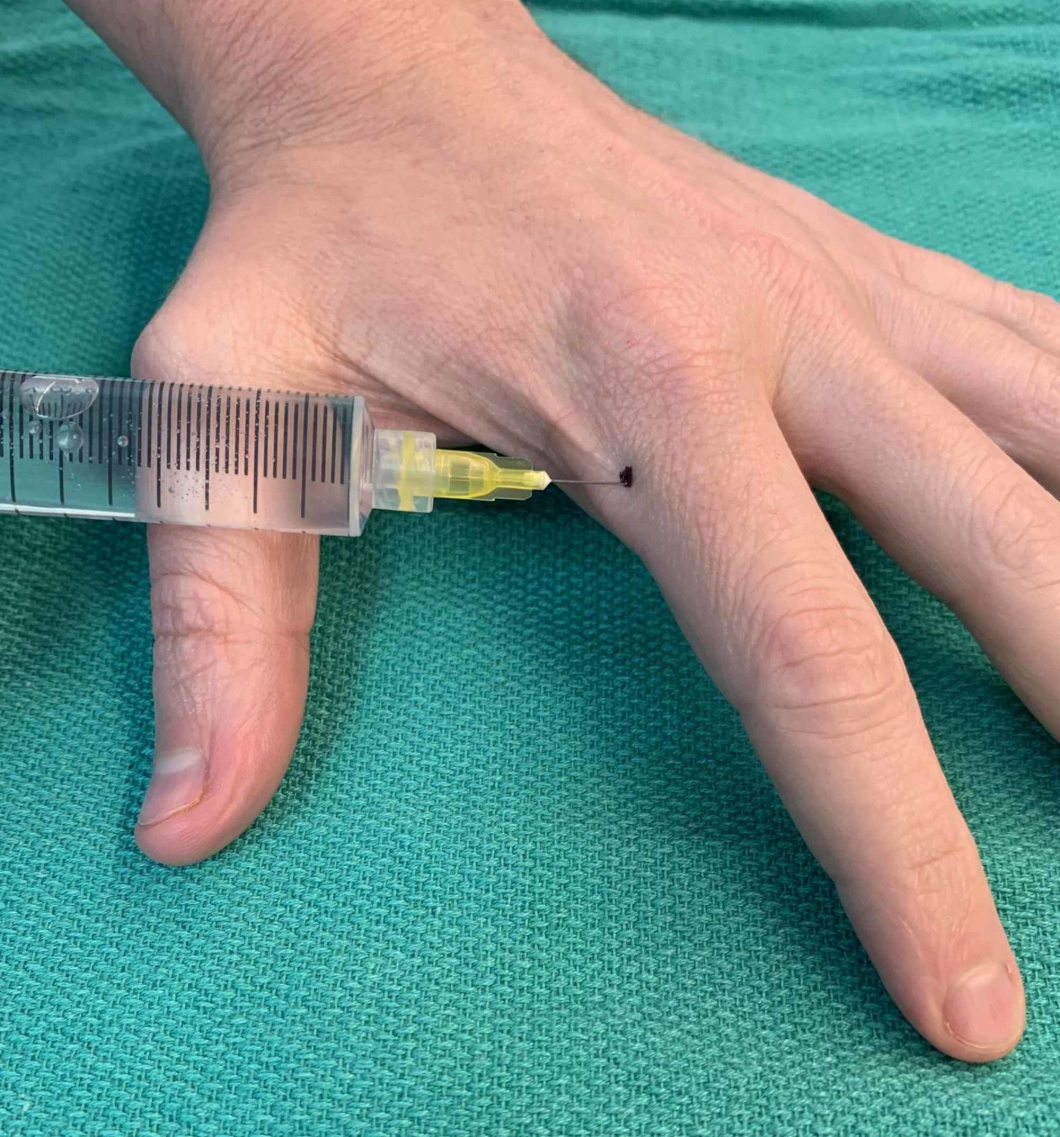
Demonstration of injection technique at the base of the second digit on the radial side (left hand)

**Figure 8:**
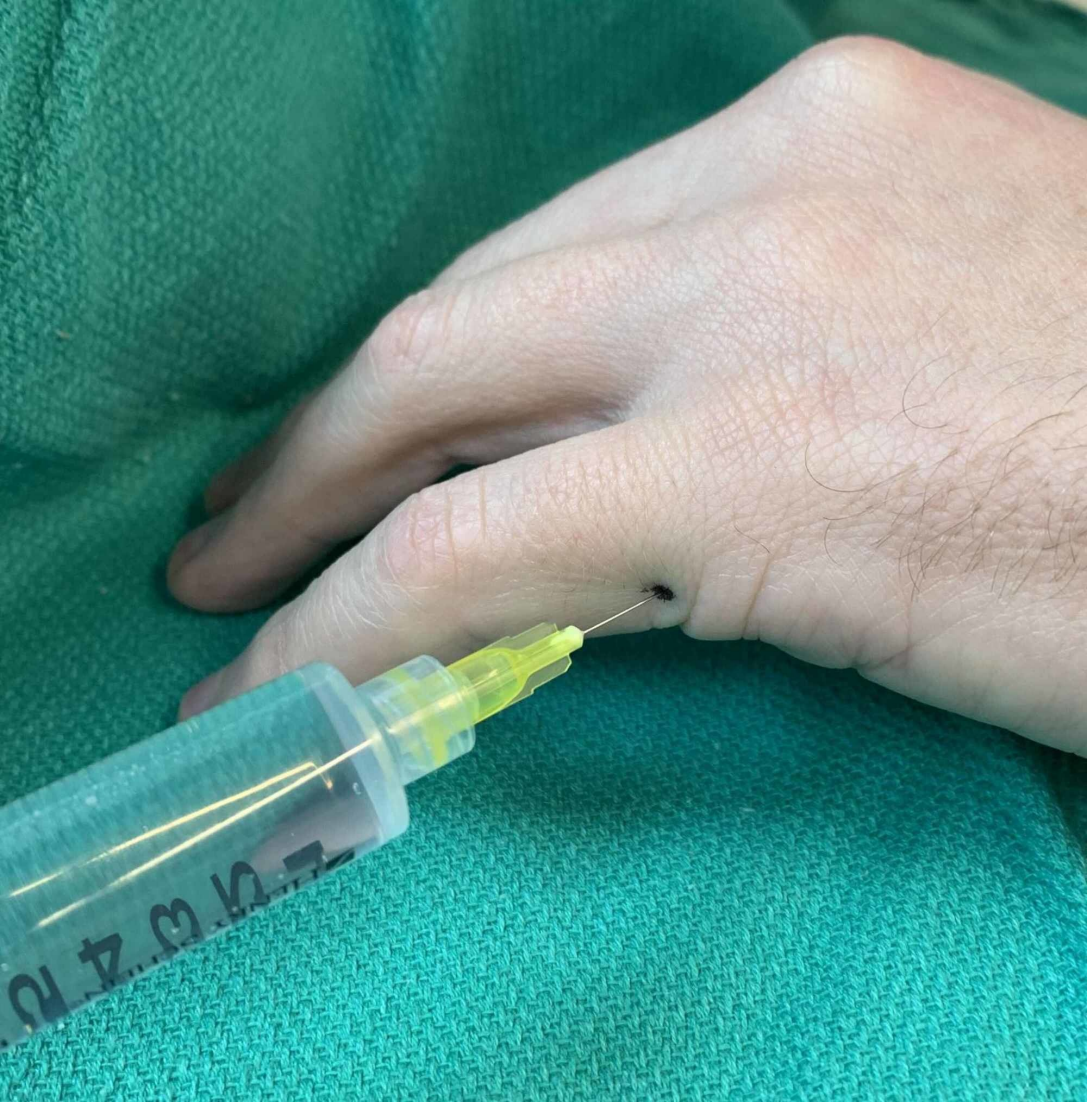
Demonstration of injection technique at the base of the second digit on the radial side (left hand)

**Figure 9:**
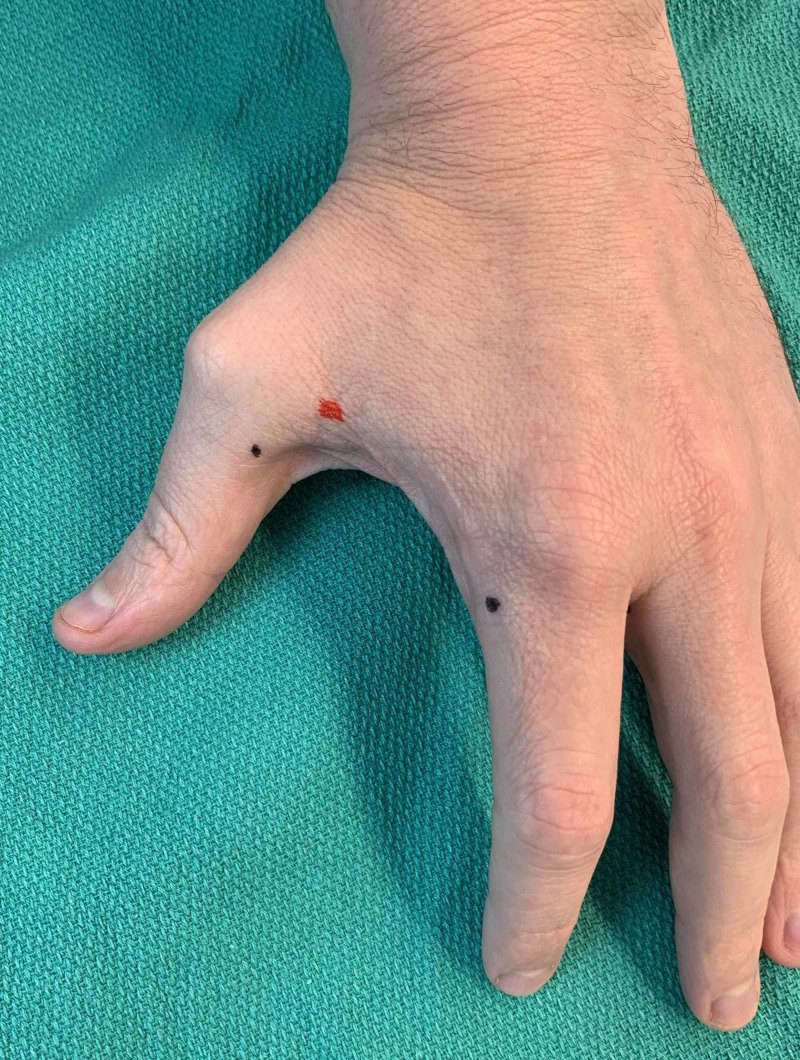
Example of correct (black dots) and incorrect (red dot) placement of injections in relation to metacarpophalangeal joints (dorsum aspect of left hand)

**Figure 10:**
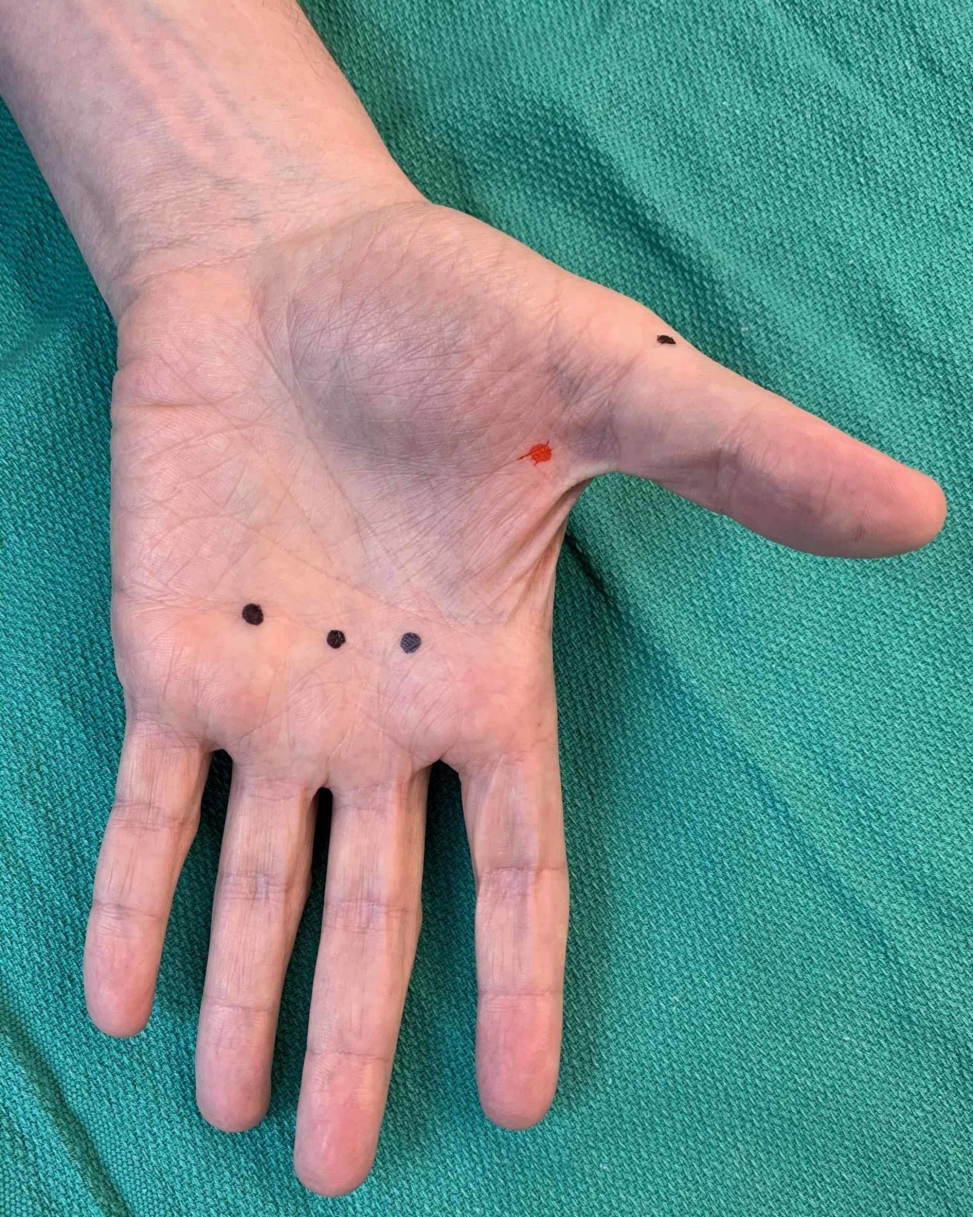
Example of correct (black dots) and incorrect (red dot) placement of injections in relation to metacarpophalangeal joints (palmar aspect of left hand)

When an entire hand was treated, 10 locations were typically injected. Three sites of injection were along the distal palmar crease between the second and third metacarpals, the third and fourth metacarpals, and fourth and fifth metacarpals in the subcutaneous space, targeting the common digital arteries (Figure 2). The three webspace injections were between the second and third digits, third and fourth digits, and the fourth and fifth digits (Figure 3). The 30-g ½” needle was fully inserted to the hub for these injections (Figure 4). The remaining four injections were located on both the radial and ulnar side of the first digit, the radial side of the second digit, and the ulnar side of the fifth digit (Figures 2, 3, 5). These injections were approximately 1-cm distal to the metacarpophalangeal joint to minimize diffusion to the thenar and hypothenar muscles (Figures 6-10). These injections were in the subcutaneous space at the junction of palmar and dorsal skin.

**Figure 11:**
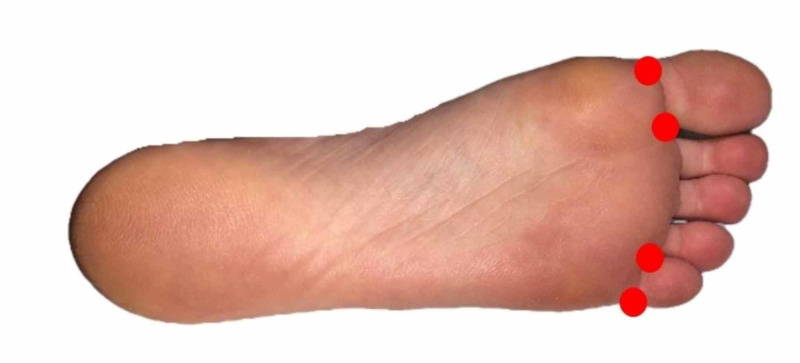
Example of injection sites as denoted by red dots (plantar aspect of left foot)

Injections of the feet and toes were administered in a similar fashion, but injections along the plantar crease proximal to the metatarsal phalangeal joints were not performed to avoid any weakness of the intrinsic foot muscles and the consequent effect on ambulation (Figure 11). If only a single or a few digits required treatment, then the ulnar and radial sides of the affected digits were injected as described above, and the distal palmar crease injections were foregone. All injections were performed by a board-certified dermatologist (DW) using aseptic technique.

If patients did not demonstrate any improvement of symptoms after two weeks, they would be seen again to receive additional treatment. Otherwise, follow-ups would be scheduled as needed.

Patient 1

A 68-year-old female with secondary RP without ulceration associated with limited cutaneous systemic sclerosis and a history of hypothyroidism, gastroesophageal reflux disease/Barrett’s esophagus, pulmonary fibrosis, supraventricular tachycardia, and hyperlipidemia reported no pain but a subjective feeling of “scaly and rough skin” in her bilateral hands. She did not desire to be on systemic medication for RP and instead was interested in localized treatment with BoNT. She received a total of 300 units of abobotulinum toxin A diluted with 10 ml of normal saline injected into 10 sites in her right hand at first to see how she tolerated the treatment. After treatment, she noted significant hand weakness that resolved after approximately six weeks. She reported a qualitative improvement in the skin on her right hand at both her nine- and 12-month follow-up visits (Table 1).

Patient 2

A 39-year-old female with RP without ulceration and a history of systemic lupus erythematosus, migraines, and rosacea received a single treatment of a total of 300 units of abobotulinum toxin A diluted with 10 ml of normal saline in her right hand. At baseline, she reported a VAS pain level of 3.5 out of 10 due to her RP and two Raynaud’s attacks per week. She also noted hand weakness that lasted six to eight weeks. By her final 12-month follow-up, she noted a 1 out of 10 on the VAS pain assessment and “overall significant improvement in her symptoms” (Table 1).

Patient 3

A 42-year-old female with secondary RP and a history of Sjögren’s syndrome, systemic lupus, limited cutaneous systemic sclerosis, rheumatoid arthritis, and autoimmune hepatitis reported pain of 8 out of 10 on the VAS for pain. Despite treatment with nifedipine and sildenafil, she still had inadequate control. She received an initial injection of 50 units of onabotulinum toxin diluted in 5 ml of normal saline to her right hand with moderate improvement, but she experienced persistent pain of the right middle and index fingers after five days. An additional 30 units of onabotulinum toxin in 3 ml of normal saline was then injected in three sites in the surrounding webspaces with subsequent improvement and resolution of pain (Table 1).

Five months later, she received 300 units of abobotulinum toxin diluted in 10 ml of 1% lidocaine over 10 injection sites on her right hand. This was repeated every three months for two more visits, typically reducing her pain level from 8 out of 10 to 2 out of 10 within a week. However, the patient noted a weakness in her right hand. At her 14-month follow-up, an ulcer was noted on the right middle finger; hence, the right hand was treated with only 240 units of abobotulinum toxin in order to minimize weakness. Once again, her symptoms resolved, and relief persisted and lasted for 10 months at which time another treatment was repeated with 270 units of abobotulinum toxin to treat her RP pain (Table 1). With this lower concentration, the patient reported equal symptom improvement and less hand weakness after three months compared to the treatment with 30 units/1 ml. At her 17-month follow-up, she had developed symptoms in her left hand and was treated with 240 units of abobotulinum toxin A. After that treatment, she noted significant improvement in symptoms, but not to the degree that she had previously noted with 300 units on her right hand. At the 21-month follow-up, she was again treated, but with 270 units of abobotulinum toxin that was diluted with 5 ml of lidocaine in order to limit diffusion, and this resulted in significant improvement (Table 1).

Patient 4

A 65-year-old female with secondary RP and a history of systemic sclerosis who was being treated with nifedipine, sildenafil, and topical nitroglycerin was referred for treatment of recalcitrant RP. She received an initial injection of 300 units of abobotulinum toxin A diluted with 10 ml of 1% lidocaine solution at 10 different injection sites to the left hand, which led to an improvement of pain and skin induration, but she noted moderate weakness. At her two-month follow-up, she received 300 units of abobotulinum toxin A (30 units/1 ml) to her right hand and again had improvement in pain but also a moderate weakness. Four months later, the patient noted RP symptoms in the toes of her left foot. She received a total of 210 units of abobotulinum toxin (30 units/1 ml) at six injection sites in the webspaces of the toes as well as to the lateral fifth toe and medial first toe (Table 1). One month later, she reported no pain in her toes as well as no RP flare-up in her hands or feet until her nine-month and 13-month follow-ups, respectively. At those times, injections of 120 units of abobotulinum toxin were administered to each of the left thumb and right ring finger over four sites each (Table 1). Her RP symptoms improved, but she was treated for a flare-up six months later in her right index finger with 60 units/1 ml over two injection sites. At her 18-month follow-up, she was having pain in her left great toe and received 45 units total of abobotulinum toxin over two sites, which reduced her RP symptoms for at least six months as of the writing of this manuscript. At her 20-month follow-up, she experienced symptoms in her right index finger and left the fifth toe, which were treated with 120 units and 60 units, respectively. The right index finger needed an additional 60 units of abobotulinum toxin six weeks later, and both digits experienced no return of symptoms for at least four months (Table 1).

## Discussion

Our four patients with secondary RP had underlying conditions of rheumatoid arthritis, systemic lupus erythematosus, Sjogren’s syndrome, and scleroderma. These patients were unable to manage their RP symptoms conservatively with both local and/or systemic conventional treatments. Hence, BoNT was considered the next step in the management of their symptoms. Multiple studies support the benefits of BoNT’s off-label use as a treatment for RP, though all of these studies have been performed with onabotulinum toxin A [6,12-14]. A 2014 study by Neumeister et al. showed onabotulinum toxin A not only improved blood flow in the hand but also reduced pain and improved function [14]. However, studies have used variable dosing regimens with differing results; nonetheless, they have almost always been positive [7,9,13-17]. A wide variety of dilutions and dosages of BoNT have been used in the treatment of RP. Dilutions ranged from 2 to 20 ml of saline for 50-100 units of onabotulinum toxin A with dosages ranging from 10 to 100 units per hand (Table 2).

**Table 2:** Summary of treatment preparation and methods from prior studies *Not mentioned in the manuscript

Study	Concentration (units/ml)	Diluent	Number of injection sites	Units per site	Total units per hand	Frequency of treatment (range)	Average follow-up time
Fregene et al., 2009 [3]	50	2.0 ml of normal saline solution	2-4	*	10-100	*	18 months
Neumeister et al., 2009 [18]	5	20 ml of normal saline solution	1-4	*	50-100	One treatment set at baseline; 4 patients (21%) required 2-4 additional injections	13-59 months
Uppal et al., 2014 [8]	20	5 ml of normal saline solution	10	10	100	One treatment set at baseline	6 months
Motegi et al, 2016 [7]	20	2.5 ml of normal saline solution	2	5	10	One treatment set at baseline	16 weeks
Wang et al., 2016 [1]	25	4 ml of 0.9% sodium chloride	9	2.5	22.5	One treatment set at baseline	2 months
Bello et al., 2017 [9]	20	2.5 ml of sterile saline solution	7	5-10	50	One treatment set at baseline	4 months
Medina et al., 2018 [13]	20	5 ml of 0.9% saline serum	8	4-8	32-64	One treatment set at baseline	3 years

While most patients show an improvement of RP symptoms with BoNT therapy, patients with long-standing RP and diffuse cutaneous systemic sclerosis may show a less significant improvement [9].

Overall, our patients were able to go months to even over a year without needing additional injections to treat their symptoms. Patients 1 and 2 reported improvements persisting over a 12-month period with only a single treatment of 300 units of abobotulinum toxin. Patients 3 and 4 reported similar results but required injections to treat intermittent exacerbations in their fingers and toes. Though none of our patients approached the recommended maximum dose of 1,000 units for abobotulinum toxin A, care needs to be taken when treating patients that have RP affecting bilateral hands and feet so as not to exceed this maximum.

Previous studies have demonstrated the efficacy of BoNT in treating patients with active ulcers or impending digits lost from RP [7,13,18]. In our series of patients, we have seen utility in using BoNT as an adjunct therapy for controlling pain, reducing the frequency of episodes, and improvement in skin quality, such as decreased induration, while still continuing with their current treatment regimen. BoNT has been of great utility when the addition of other systemic pharmacologic therapies is limited by factors such as hypotension.

We frequently use lidocaine as opposed to normal saline to reconstitute the BoNT not only to decrease injection pain but also because of its vasodilatory properties [19]. It is possible that some of the benefits that our patients reported could be attributed to lidocaine, though it is unlikely due to its half-life of 90-120 minutes. In our experience, patients are often in moderate to severe pain when they come in for treatment and the use of lidocaine contributes to pain relief, though short-lived. When using lidocaine to reconstitute BoNT for use in patients with RP, it is imperative that the lidocaine does not contain epinephrine as this could induce a flare/episode with a potential loss of digits.

Hand weakness is commonly encountered when treating RP of the hand with BoNT with studies reporting 16-27% incidence [14]. Previous studies on BoNT for palmar hyperhidrosis report that 30-50% of patients treated experienced weakness [20]. This can sometimes outweigh the benefit of Raynaud’s symptom improvement as the weakness in two patients in our study was noted to interfere with routine tasks such as turning the key in the ignition of the car or opening a jar of peanut butter. We found that placing the injections around the thumb just distal to the metacarpophalangeal joints resulted in significantly less weakness in our patients. Other reported complications include transient dysesthesia (4% in a single study) and bruising [14,20]. It also appears that it is difficult to predetermine the dosage and frequency needed to alleviate symptoms and they vary from patient to patient. Thus, future studies with a larger sample size are needed to examine dose frequency as a variable in treating patients with secondary RP.

As the treatment is not FDA-approved for RP, insurance is unlikely to cover the procedure, leading to high out-of-pocket costs. Insurance coverage is challenging, always requiring prior authorization and going through an appeals process. Even then, there is still no guarantee of coverage. We have found that sending before and after clinical photos with the appeal documentation has been helpful in obtaining coverage of treatments. The procedure is billed with CPT code 64999 (unlisted procedure, nervous system) and J code J0586 (injection, abobotulinumtoxinA, 5 units) or J0585 (injection, onabotulinumtoxinA, 1 unit), depending upon which type of BoNT is used. Most manufacturers of BoNT have patient assistance programs that can reduce the cost of the medication. When treatment is not covered, the medication is billed at cost to the patient, and the visit is billed as a standard E/M visit to their insurance.

## Conclusions

Secondary RP is a debilitating disease for many patients. Current research supports the need for further investigations and clinical use of BoNT as an alternative therapy for patients who cannot control their symptoms through conservative and pharmacological therapies alone. We reported the case of four patients who experienced clinical improvement with both singular and multiple BoNT injections. Moreover, it appears that higher doses of BoNT are viable treatment options for patients with secondary RP.
